# Functional neurological disorder is a feminist issue

**DOI:** 10.1136/jnnp-2022-330192

**Published:** 2023-03-28

**Authors:** Caoimhe McLoughlin, Ingrid Hoeritzauer, Verónica Cabreira, Selma Aybek, Caitlin Adams, Jane Alty, Harriet A Ball, Janet Baker, Kim Bullock, Chrissie Burness, Barbara A Dworetzky, Sara Finkelstein, Béatrice Garcin, Jeannette Gelauff, Laura H Goldstein, Anika Jordbru, Anne-Catherine ML Huys, Aoife Laffan, Sarah C Lidstone, Stefanie Caroline Linden, Lea Ludwig, Julie Maggio, Francesca Morgante, Elizabeth Mallam, Clare Nicholson, Mary O’Neal, Suzanne O‘Sullivan, Isabel Pareés, Panayiota Petrochilos, Susannah Pick, Wendy Phillips, Karin Roelofs, Rachel Newby, Biba Stanton, Cordelia Gray, Eileen M Joyce, Marina AJ Tijssen, Trudie Chalder, Maxanne McCormick, Paula Gardiner, Indrit Bègue, Margaret C Tuttle, Isobel Williams, Sarah McRae, Valerie Voon, Laura McWhirter

**Affiliations:** 1 Centre for Clinical Brain Sciences, University of Edinburgh, Edinburgh, UK; 2 Neurology Department, Centro Hospitalar Universitário de São João, Porto, Portugal; 3 Department of Clinical Neuroscience, Hopitaux Universitaires de Geneve, Geneva, Switzerland; 4 Department of Clinical Neuroscience, Inselspital Universitatsspital Bern Universitatsklinik fur Neurologie, Bern, Switzerland; 5 Department of Psychiatry, Massachusetts General Hospital, Boston, Massachusetts, USA; 6 Massachusetts General Hospital, Harvard Medical School, Boston, Massachusetts, USA; 7 College of Health and Medicine, University of Tasmania, Hobart, Tasmania, Australia; 8 Neurology, Royal Hobart Hospital, Hobart, Tasmania, Australia; 9 Bristol Medical School, University of Bristol Faculty of Health Sciences, Bristol, UK; 10 Neurology, North Bristol NHS Trust, Westbury on Trym, UK; 11 Randwick Specialist Centre, Private Practice, Randwick, New South Wales, Australia; 12 College of Nursing and Health Sciences, Flinders University, Adelaide, South Australia, Australia; 13 Department of Psychiatry and Behavioral Sciences, Stanford School of Medicine, Stanford, California, USA; 14 Neurology, Walton Centre NHS Foundation Trust, Liverpool, UK; 15 Department of Neurology, Brigham and Women's Hospital, Harvard Medical School, Boston, Massachusetts, USA; 16 Department of Neurology, Massachusetts General Hospital, Boston, Massachusetts, USA; 17 Department of Neurology, Hopital Avicenne, Bobigny, France; 18 Department of Neurology, Vrije Universiteit Amsterdam, Amsterdam, The Netherlands; 19 Department of Psychology, Institute of Psychiatry, Psychology and Neuroscience, King’s College London, London, UK; 20 Faculty of Humanities, Sport and Educational Science, University of South-Eastern Norway, Kongsberg, Norway; 21 Department of Neurology, Cambridge University Hospitals NHS Foundation Trust, Cambridge, UK; 22 Neurology, St. James’s Hospital, Dublin, Ireland; 23 University Health Network and the University of Toronto, Toronto, Ontario, Canada; 24 Department of Health, Ethics and Society, Faculty of Health, Medicine and Life Sciences, Maastricht University, Maastricht, The Netherlands; 25 Department of Clinical Psychology and Psychotherapy, University of Hamburg, Hamburg, Germany; 26 Department of Physical Therapy and Functional Neurological Disorder Unit and Research Program, Massachusetts General Hospital, Boston, Massachusetts, USA; 27 Neurosciences Research Centre, Molecular and Clinical Sciences Research Institute, St George's University Hospitals NHS Foundation Trust, London, UK; 28 Department of Experimental and Clinical Medicine, University of Messina, Messina, Italy; 29 The Rosa Burden Centre, Southmead Hospital, North Bristol NHS Trust, Bristol, UK; 30 Therapy Services, National Hospital for Neurology & Neurosurgery, University College London Hospitals NHS Foundation Trust, London, UK; 31 Neurology, National Hospital for Neurology and Neurosurgery, London, UK; 32 Movement Disorders Program, Neurology Deparment Hospital Ruber Internacional, Madrid, Spain; 33 Movement Disorders Unit, Neurology Department, Hospital Ramón y Cajal, Madrid, Spain; 34 University College London Hospitals NHS Foundation Trust, London, UK; 35 Section of Cognitive Neuropsychiatry, King's College London, Institute of Psychiatry, Psychology and Neuroscience, London, UK; 36 Donders Institute for Brain Cognition and Behaviour: Donders Centre for Cognitive Neuroimaging, Radboud University Nijmegen, Nijmegen, The Netherlands; 37 Behavioural Science Institute, Radboud University Nijmegen, Nijmegen, Netherlands; 38 Neurology, Teaching Hospitals NHS Foundation Trust, Sheffield, UK; 39 Neurology, King's College Hospital, London, UK; 40 Neurology Psychotherapy Service, Sheffield Teaching Hospital, Academic Neurology Unit, The University of Sheffield, Sheffield, UK; 41 Neuropsychiatry, UCL Queen Square Institute of Neurology, London, UK; 42 Expertise Center Movement Disorders Groningen, University Medical Centre Groningen, Groningen, The Netherlands; 43 Department of Psychological Medicine, Institute of Psychiatry, Psychology and Neuroscience, Kings College London, London, UK; 44 Physician assistant/patient with FND, FNDRecovery.com, -, Monument CO, USA; 45 Psychological Therapy in Primary Care, University of Dundee, Dundee, UK; 46 enhance-cbt.com therapist, NeuroSpecialist Physiotherapist, Stirling, UK; 47 Department of Psychiatry, Geneva University Hospitals, Geneve, Switzerland; 48 Department of Psychiatry, Functional Neurological Disorder Unit, Massachusetts General Hospital, Boston, Massachusetts, USA; 49 Neuropsychology, Department of Clinical Neurosciences, NHS Lothian, Edinburgh, UK; 50 Department of Clinical Neurosciences, NHS Lothian, Edinburgh, UK; 51 Psychiatry, University of Cambridge, Cambridge, UK; 52 Institute of Science and Technology for Brain-Inspired Intelligence, Fudan University, Shanghai, China

**Keywords:** conversion disorder, functional neurological disorder, neuropsychiatry, somatisation disorder

## Abstract

Functional neurological disorder (FND) is a common and disabling disorder, often misunderstood by clinicians. Although viewed sceptically by some, FND is a diagnosis that can be made accurately, based on positive clinical signs, with clinical features that have remained stable for over 100 years. Despite some progress in the last decade, people with FND continue to suffer subtle and overt forms of discrimination by clinicians, researchers and the public. There is abundant evidence that disorders perceived as primarily affecting women are neglected in healthcare and medical research, and the course of FND mirrors this neglect. We outline the reasons why FND is a feminist issue, incorporating historical and contemporary clinical, research and social perspectives. We call for parity for FND in medical education, research and clinical service development so that people affected by FND can receive the care they need.

## Introduction

Some women get erased a little at a time, some all at once. Some reappear.—Rebecca Solnit

Following centuries of neglect, gender and sex discrimination in healthcare now receives more widespread attention; but despite improved awareness, inequality remains common in biomedical settings. Research funding is disproportionately directed towards the investigation of diseases that primarily affect men at the expense of those that affect women.^1 2^ Clinically, gender bias affects the management of medical complaints such as dizziness, pain or fatigue, with women less likely to receive appropriate diagnostics, treatment or follow-up.^3 4^ The mortality rate in cardiovascular disease is much higher in women than in men, but diagnostic criteria and treatment thresholds generally do not take account of sex or gender.^5^ Female participants have historically been under-represented in clinical studies and were essentially excluded from clinical trials up until the 1990s.^6 7^ These inequalities are not unique to the scientific realm. Women suffer disproportionately from the health effects of violence, poverty and social exclusion—this is a global problem.

The impact of implicit biases on the basis of sex and gender can be seen in the lack of recognition of marginalised, stereotypically ‘female’ medical disorders such as chronic fatigue syndrome/myalgic encephalomyelitis, anorexia nervosa and migraine.^2^ We argue that functional neurological disorder (FND) is similarly marginalised.

FND is a common and disabling disorder presenting with a variety of clinical manifestations, including weakness, sensory changes, involuntary movements, gait disturbance, dissociative episodes and speech problems. It is described as a multinetwork disorder involving abnormalities within and across brain circuits implicated in emotion processing, attention, interoception, speech, motor control and sense of agency, among other functions.^8^ FND is the second most common reason for patients to attend neurology clinics, estimated to have an incidence of 4–5 per 100 000 and therefore to be present in the population at a similar frequency to multiple sclerosis and Parkinson’s disease.^9 10^ Women are disproportionately affected by FND across phenotypes, with rates of 70% in most large studies.^11^

Despite this, people with FND seeking treatment often wait years to receive treatment, with some patients waiting a mean time of 8 years before the diagnosis is made.^12^ The road to diagnosis for patients with FND is often marred by misunderstanding, fruitless investigations and a sense of being ‘passed from pillar to post’. People with FND have similar or worse levels of distress and disability than those with other neurological conditions such as multiple sclerosis or epilepsy.^9^ Without treatment, few patients recover, and delayed diagnosis results in worse long-term outcomes.^13^ Yet there is potential for improvement and resolution of symptoms even in patients with longstanding FND; small treatment trials for motor FND have demonstrated significant improvements in quality of life and physical function.^14 15^ In addition to being detrimental to patients’ lives, leaving patients undiagnosed with FND incurs major costs for governments, and health and social care systems.^16^

Much has been written about the history of FND, some of which includes themes of maltreatment of women.^17^ The terms used in these historical descriptions vary—hysteria, for example, is used as a broad classifier that encompasses heterogeneous presentations, some which would be recognisable as FND to a modern reader. This was a diagnosis much more frequently applied to women than men, and some sociologists and scientists have opined that the diagnosis was used as a ‘patriarchal tool’ to silence or ignore complaints of women.^18^

We, a group of clinicians and academics who research and treat patients with FND, recognise that many people do feel ignored or dismissed on receipt of an FND diagnosis. Some perceive that the diagnosis of FND has been used euphemistically to denote an illness that is imagined, or worse, feigned.^19^ Labels such as ‘psychogenic’ or ‘pseudo’ may have been used to dismiss or deny the disabling impact of symptoms and may arguably be used as a reason for inaction from healthcare professionals. That said, when appropriately diagnosed, we do not agree that FND is any longer a tool used to silence women or dismiss their symptoms. Rather, we propose that the discrimination and harm which people with FND experience—and which disproportionately affects women —is the result of chronic uninterest in and neglect of FND by neuroscience and healthcare establishments.

We are a group of women clinicians and academics, and we believe that people of all gender identities, and the conditions which affect them, should be equally respected and have parity of access to funds for investigation and treatment. Building on a core definition of feminism as the belief that women should be afforded the same rights, power and opportunities as men and be treated in an equitable way, we further note clinical ethicist Professor Wendy Roger’s view that “… a feminist approach to health inequities leads us to examine the connections between disadvantage and health, and the distribution of power in the processes”.^20^ In this paper, we discuss the epidemiology, diagnostic stability and current theoretical understanding of FND, and explain why seeking equity in care and research for people with FND is a feminist issue.

### Stigma and misperceptions surrounding FND

Stigma is pervasive in FND and occurs in many forms, subtle and overt. The origins of this stigma are complex and may arise from issues around FND’s associations with psychiatric diagnoses and misperceptions of the degree of voluntary control held by patients. This mirrors the stigma seen in other functional disorders such as fibromyalgia, and psychiatric diagnoses such as emotionally unstable personality disorder, and indeed in depression and anxiety disorders—all diagnoses that are more prevalent in women than men.^21 22^ Historically, the interplay of women’s health and societal factors has been studied in great depth, particularly in the Victorian depictions of neurasthenia and hysteria, where there are multiple examples of female characters being portrayed as emotionally labile and unpredictable, in contrast with their more rational and contained male counterparts, who hold more power.^23^

Undoubtedly, progress has recently been made. However, people with FND continue to describe experiences of doubt, blame and of being seen as less ‘genuine’ than those with other disorders, particularly ones with more obvious structural pathology.^19^ This is perpetuated inter-generationally by FND being somewhat disregarded in undergraduate and postgraduate training, undermining its importance as one of the most common causes of neurological symptoms. Although academic interest in FND is difficult to accurately track given the inconsistent terminology used throughout decades, by 1970 it was almost completely absent from medical textbooks.^24^

Lack of undergraduate and postgraduate education in FND means that well-intentioned junior clinicians are vulnerable to the repeated hits of the ‘hidden curriculum’.^25^ Outdated perceptions might be passed down from senior clinicians (often male) to junior clinicians (currently more mixed). For example, patients are often referred to as ‘heartsinks’ or ‘time wasters’. While we cannot directly control the hidden curriculum, ensuring focus on FND early in medical education could potentially encourage openness to new ways of thinking and resistance to old perceptions and prejudices, thus breaking the cycle of misinformation which has such a detrimental impact on patient care.

These old-fashioned but ever present and stigmatising attitudes are further fuelled by misunderstanding around the aetiology and presentation of FND. In our experience, clinicians, patients and caregivers raise the following tropes about FND that can perpetuate stigma: (1) FND represents a missed alternative diagnosis; and (2) FND is thought of as arising from a disembodied ‘mind’ in an extreme dualistic model of the mind as separate from the body.^26 27^ This stigma is important to acknowledge and describe, as it provides an important context to the more nuanced gender-based inequity we see in FND.

To address the first point, although we cannot ignore occasional misuse of the term, FND diagnoses when made appropriately are stable, reliable and unlikely to represent a missed alternative diagnosis. Descriptions of clinical presentations of the disorder we presently call FND are remarkably stable geographically and historically.^10 28 29^ This is an important point to highlight because despite a waxing and waning interest by clinicians and researchers, the clinical presentation of FND does not seem to have changed.

Diagnoses of FND, just like those of migraine, Parkinson’s disease or motor neuron disease, are primarily made in the clinic, on the basis of reproducible history or signs, before imaging or other investigations are undertaken. Signs which have been proven to be reliable in motor FND include Hoover’s sign, hip abductor sign, drift without pronation, identification of typical FND gait, and ipsilateral weakness of the sternocleidomastoid with hemiparesis.^14 30–32^ In functional (dissociative) seizures, the clinical signs of active resistance to eye opening, flutter or blinking on eyelash rub, or change in the seizure semiology in response to others during an episode have 100% specificity.^33–35^

Physicians seem to fear giving an incorrect diagnosis. But in a review of 27 studies with FND, with a total population of 1466, the proportion of misdiagnosis was less than 4% after an average of 5 years of follow-up.^36^ Even after lengthy follow-up, the diagnosis remains stable—a recent 14-year follow-up study described a diagnostic revision rate of 1%.^37^ Indeed, misdiagnosis occurring in the opposite direction can be quite catastrophic, for example, if a person with FND is diagnosed with multiple sclerosis, epilepsy or dementia, substantial suffering may ensue due to incorrect treatments or prognoses being offered. Inaccurately diagnosing functional seizures as epileptic seizures can result in high doses of medications, intubation and intensive care unit admissions, with the associated risks including death.^38^ It has been shown that costs per admission for FND are increasing at a higher rate than that of other neurological disorders, with iatrogenic harm and inappropriate investigations likely inflating costs.^16^

To address the second point, that FND lacks validity on the basis that symptoms are feigned or imagined, there is simply little evidence of this. The persistence of positive signs used to identify FND such as contractures in patients with fixed dystonia, persistence of Hoover’s sign even after the patient has been shown how it operates, evidence of shoe wear in patients with functional gait disorders, stability in presentation and improvement with therapy are some of the many features that indicate FND is not compatible with feigning.^39^ Behavioural neuroscience studies have clearly demonstrated that sensorimotor disturbance in FND differs from that of healthy controls in a manner that is not compatible with voluntary feigning.^40 41^

Furthermore, functional and structural changes in the brains of people with FND have demonstrated evidence of increased connectivity between motor control and emotional processing areas.^40 42–44^ These laboratory findings support a conceptual understanding of FND as a result of glitches in the brain’s predictive processing system. The brain is thought to represent Bayesian network, with prior experiences and beliefs assembled to form predictive models (‘top down’ sources of information) about the world. This allows rapid responses to an ever-changing environment. Sensory information (‘bottom up’) is constantly being fed into this predictive model to test and enhance it in order to minimise prediction error. This allows for adaptive responses to changing environmental circumstances. However, the ‘top down’ prediction weighting can be increased by attentional focus, mediated by the salience network and limbic system.^45 46^ In FND, it is hypothesised that abnormal predictions about movement, strengthened by abnormal self-directed attention overwhelm sensory evidence to generate movements that have not been consciously planned, and thus occur without a normal sense of agency (volitional control).^47^ Evaluating these models, FND challenges dualistic notions of brain and mind.

This brings us to our third point; FND cannot be assigned to a simple psychological or physical category. Simply put, our brain is a complex organ, responsible for emotions, attention, movement, sensations and predictions about the world. All of these aspects exert influence on each other and are shaped by our experiences—be it life events, neurological illness or something else we cannot yet identify. It is impossible to disentangle these aspects of functioning from each other. Dated ‘conversion’ theories, that trauma is always the underlying cause of functional symptoms, are too simplistic, often do not make sense to patients and have rightly been removed as essential criteria from international classifications.

This is not to undermine the importance of life events in the aetiology of FND. A recent systematic review and meta-analysis found maltreatment was substantially more common in people with FND than in healthy controls and patient controls.^48^ While there are certainly patients for whom trauma is not relevant, studies have consistently shown that violence and sexual abuse, particularly childhood abuse, are aetiologically and prognostically significant in FND.^48–51^ These are issues that, worldwide, disproportionately affect women.^52^ These gender-weighted risk factors will be discussed further.

### Why FND is a feminist issue

We lived in the gaps between the stories—Margaret Atwood, *The Handmaid’s Tale*

FND is a feminist issue. We say this because (1) FND predominantly affects women; (2) historical and societal issues affecting women continue to shape the narrative of FND; (3) under-recognition of FND occurs in men due to potential diagnostic bias; (4) sexual abuse and violence are gender-weighted risk factors for FND; (5) socioeconomic disparity exists between men and women, contributing to inequalities in access to treatment; (6) FND clinical services and research are chronically underfunded, in line with the neglect of disorders disproportionately affecting women.

#### Historical narrative

Functional disorders, including FND, have a problematic history. These conditions, among other disorders originating from the brain, such as epilepsy and psychotic disorders, were historically drowned in prejudice and even punishment; and what would be described today as FND has been depicted in terms of moral failing, demonic possession, hysteria or witchcraft; with uterine repositioning reported to be a proposed treatment.^53^ The iconic patients in La Salpêtrière Hospital in Paris (most of whom were women) have been well studied from a neurological standpoint; however, we hear little about the deplorable conditions of extreme poverty and male subjugation that these women came from, or of the objectification and exploitation they were subject to on admission and afterwards.^17 54^

These accounts of treatment, while extreme, unfortunately parallel accounts given by patients with FND today. We continue to hear of patients with FND being ‘shamed, blamed and humiliated’ on account of their diagnosis.^55^ Particular issues that are relevant for female patients with FND have received little attention; for example, functional seizures are typically diagnosed in women of childbearing age; however, it is not uncommon for these women to be prescribed potentially teratogenic anti-seizure medications.^56^ There is minimal literature available describing cases and treatment of FND in pregnancy^57–60^ and this gap in the literature needs to be urgently addressed, given the risks to woman and child.

The prejudicial treatment of women with functional disorders is evident in a recent longitudinal study by Ballering and colleagues, describing the management of persistent somatic symptoms, most of which were likely functional in origin. They showed that women presenting with symptoms of dizziness, tiredness, pain and tingling were less likely to receive a physical examination, diagnostic imaging and specialist referral for their complaints than men. They were also less likely to receive a clear diagnosis for their symptoms.^3^

However, historical biases may make physicians more likely to diagnose FND in women than in men, despite similar symptom profiles.^61^ This longstanding bias is exemplified in the different terminology used to describe symptoms of ‘hysteria’ in women and men. Showalter discusses how the concept of hysteria represented an unwanted fragility that was unacceptable for men, “hysteria in men has always been regarded as a shameful, ‘effeminate’ disorder”.^18^ For men and their (mainly male) doctors, the diagnosis was concealed beneath a fabric of alternative descriptors, such as ‘neurospasme’, hypochondria or shellshock.^18 62^ Neurasthenia—a condition similar in many ways to hysteria—was seen as an affliction of the ‘male elite’, caused by the repercussions of productive life such as ‘overwork, sexual excess and ambition’, a contrast to the pejorative female counterpart.^18^ Newer descriptors have since emerged, such as ‘psychogenic’, ‘conversion disorder’ and now functional— however, despite changes in terminology, the stigma remains. In contrast, neurasthenia, shellshock and hypochondria—the more ‘male disorders’ became associated with less stigmatising terminology (eg, post-traumatic stress disorder, health anxiety) categorically distancing from what is now FND, distancing from the associated prejudice too. The term ‘hysteria’ was removed from the third version of the Diagnostic and Statistical Manual of Mental Disorders in 1980.

Yet against this backdrop of changing terminology and prejudice, careful analysis of historical case records tells us that clinical presentations of FND (in both women and men) have in fact remained remarkably consistent over the last century.^63^

#### Gender bias in diagnosis

Briquet, a French physician, was one of the first to describe and support the diagnosis of hysteria in men, although he did not agree with the use of the term hysteria.^64^ This work was continued by Charcot, who similarly pointed out that hysteria was certainly observable in men, at the time a contentious finding.^65^

There is a large amount of data suggesting that FND affects more women than men.^11 66 67^ Current estimates tend towards a female to male sex ratio of 2–3:1.^10^ 10 Estimates for functional voice disorders, although based on smaller series, suggest a potentially higher ratio of 8:1.^68^ However, a large recent study looking at dissociative seizures indicated that the proportion of men with dissociative seizures increased with age at onset.^67^ These findings replicate those of Duncan *et al* 69 who found minimal sex differences in frequency of dissociative seizures in patients aged over 55 years, with predisposing factors such as health-related traumatic experiences more important in the older group. Similarly, late onset functional myoclonus, while more common in females, affects a substantial proportion of males.^10 70^

Future research is needed to explore the reasons for these differences—with attention to biological, psychological and social factors. While we are not disputing that FND is more common in women, as the evidence clearly demonstrates, there may be some limitations to these epidemiological figures. It is possible there may be some physician bias against diagnosing FND in men, given the way FND has been (and continues to be) portrayed and taught. Morgante *et al* found that male gender significantly impacted diagnostic agreement in functional movement disorders.^71^ Carson also pointed out that women are in general 1.5 times more likely to present to health services,^10^ so in reality FND might affect more men than we see. It would be helpful to carry out further population-based studies to ascertain the true proportions of all sexes and genders affected by FND, although pragmatically challenging given the diagnosis is primarily clinic-based.

### Gender-weighted risk factors

The elevated rates of FND in women might reflect complex differential exposures to social and environmental risk factors. Women are far more likely to have experienced childhood sexual abuse, intimate partner violence and sexual assault than men.^72 73^

A recent important study found a significant association between sexual abuse and FND in women, and a greater likelihood that women who are sexually abused will develop functional movement disorders than men who are sexually abused.^49^ Another recent study examining sex differences in functional seizures showed that reported sexual abuse, physical abuse and family dysfunction were significantly higher in females compared with males.^74^ In a meta-analysis of controlled studies, Ludwig *et al* found that life events—most commonly maltreatment in the form of neglect, physical and sexual abuse—were experienced by patients with FND eight times as frequently as healthy controls and twice as often as in other psychiatric and neurological conditions.^48^ Morsy *et al* expanded on the review, finding that family, relationship and work adverse events were also more common in patients with FND than controls—where the control group were patients diagnosed with another psychiatric or neurological condition.^50^ Of note in this study, work events were more commonly an issue for men, while family events were more common for women; such family events were often related to marital conflict or domestic discord. The association between sexual abuse and FND is further supported by another recent meta-analysis finding that among major diagnoses used in psychiatric practice, FND had the strongest association with sexual abuse.^75^

Associations between women, violence and FND span cultures and ethnicities. A large Bolivian study showed that rates of functional seizures were much higher in women who suffered psychological, sexual and physical intimate partner violence than those who did not.^76^ Latin America has the highest rate of violence against women in the world.^77^ ‘Ataque de nervios’ is a Latin American syndrome where patients suffer from symptoms similar to FND symptoms, such as dissociation and seizures. Population studies show that up to 15% of Latin American women experience this syndrome, with the risk increased by poverty, disrupted marriage and trauma.^78 79^

There are issues that, worldwide, disproportionately affect women. Estimates published by WHO indicate that globally almost 1 in 3 (30%) of women worldwide have been subjected to either physical and/or sexual intimate partner violence or non-partner sexual violence in their lifetime.^52^

There may be additional, more ‘hidden’ gender-weighted risk factors outside trauma that are important in the aetiology of FND, such as societal and familial expectations. This may be partly captured in the ‘family events’ noted in the above review by Morsy *et al*.^50^ In this study ‘family event’ is a term used in a broad sense describing household responsibilities, family dysfunction and a lack of family or social support. Although understudied in FND, being in a caregiving role is associated with higher rates of mental and physical health impairment, where again women are more adversely affected.^80 81^ COVID-19 has further highlighted the significant burden carried by women when it comes to childcare, with considerable psychosocial sequelae. Data covering 193 countries spanning all income levels showed that during the pandemic females were more likely to report employment loss than men, as well as forgoing work to take on caregiving roles. They were also more likely than males to report dropping out of school for reasons other than school closures.^82^ Lack of education and employment has a direct impact on socioeconomic status, and socioeconomic disparity has an excessive impact on women, adversely affecting their health.

Although data are lacking, we also acknowledge the likely intersection of other biases and inequalities as contributors to stigma and discrimination experienced by transgender women and non-binary people with FND.

### Socioeconomic disparity, women and FND

Women’s health status is significantly lower than men’s across the globe, and this disparity is associated with education, employment and economic status.^83^ A large proportion of people with FND come from lower socioeconomic backgrounds.^84–86^ Research comparing long-term prognosis in patients with FND and healthy controls showed levels of unemployment were very high in the FND group, at 41%.^37^ Studies reporting on FND in resource-limited countries are scarce; however, it is interesting to note that in a recent study carried out in Sudan, 60% of patients diagnosed with FND were women from lower socioeconomic backgrounds, paralleling global trends.^87^

Access to appropriate diagnostics and treatments for FND remains largely dependent on socioeconomic status, with a clear relationship demonstrated between income and access to specialist expertise for patients with FND.^88^ Economic disadvantage therefore both increases risk of FND and reduces access to specialist treatment; again, women are disproportionately impacted.

#### Underfunding in research

The situation around allocation of resources to FND research parallels that of females in research generally. Females are considerably under-represented in research. Reasons for the exclusion of female subjects in medical trials include hormonal differences, cost and lack of comparability with previous trials in solely male participants.^89^ Females of animal species also have been under-represented in biomedical studies—neuroscience being the worst culprit.^6^ Studies that do assess the influence of sex have identified that sex does matter, with significant differences between the male and female central nervous system. For example, Huang and Woolley discovered significant differences in synaptic modulation in the hippocampus and endocannabinoid tone between males and females.^90^ Stroke research reveals sex-specific factors that affect onset and outcome which are often under-recognised in women (eg, pre-eclampsia). It is not widely appreciated that women have worse outcomes, quality of life and increased disability after stroke compared with men.^91^ There are also significant sex differences in pain sensitivity and analgesia responses between men and women.^92^ But although pain disorders—common comorbidities in FND—occur more frequently in women than in men, pain research has been substantially dominated by male research participants.^93^

In an examination of allocation of funding to medical research, Mirin outlines striking disparities in funding relative to disease burden between diseases that primarily affect men, and those that primarily affect women—chronic fatigue syndrome/myalgic encephalomyelitis, migraine and other headache disorders being top of the list.^2^ While FND did not feature specifically, little scrutiny is needed to demonstrate that research funding falls far short. Despite it being one of the most common reasons for presentation to the neurology clinic, there have been few randomised controlled multicentre trials looking at treatment for FND. At the time of writing, the largest clinical trial register shows 285 studies are currently recruiting for epilepsy, 185 for motor neuron disease, 446 for multiple sclerosis and 556 for Parkinson’s disease (clinicaltrials.gov). Only 10 are currently recruiting for FND,^94^ despite it having similar rates of disability and distress to other neurological conditions.^95^

## Conclusion

Nothing I accept about myself can be used against me to diminish me.—Audre Lorde, Sister Outsider

The history of FND, in some ways, mirrors the history of women in society. It is a history laden with inequalities, dismissal and injustice which cannot be undone. Now, patients with FND do not need pity, but parity. The inequalities and injustices continue today in the form of ignorance of FND in teaching curricula, delayed diagnosis, stigmatising healthcare interactions, paucity of specialist services and an underfunding of scientific research. But discrimination is not an inevitable component to the FND diagnosis, which, when it is properly made, is as stable and accurate as that of any other common neurological disorder.

We are not the first group to highlight that FND critically requires parity of esteem with neurological conditions of equivalent epidemiological and economic importance.^96^ Patients have also now joined in this discussion. Social media has given people with FND a voice, and they have been vocal and brave in rightly echoing this need for recognition. We commend and support those who live with FND who have risen above the parapet to discuss their own experiences. We must do more than listen to these voices—we must take meaningful action.

This is a call to action. We support and urge careful and appropriate use of this diagnosis to support and empower those affected by FND so their symptoms and suffering can be recognised and validated. We call for respectful models of clinical care and an end to dismissive and harmful language and behaviour towards people with FND. We call for a shift in approach to FND, and other functional disorders, to move away from dualist models of mind and body. We call for parity for FND among other neurological disorders in medical education, and better training curricula for all the allied professions that have contact with patients with FND.

We call for more recognition and funding for more impactful laboratory and clinical research, and support of female leadership in the FND community to improve diversity and excellence in the field. We urge planning and funding for better and more universally available FND treatment services, and a universal upskilling of clinicians, so that people with FND can finally receive the treatment they require and deserve.
